# PRIMED^2^ Preclinical Evidence Scoring Tool to Assess Readiness for Translation of Neuroprotection Therapies

**DOI:** 10.1007/s12975-021-00922-4

**Published:** 2021-07-01

**Authors:** Mersedeh Bahr-Hosseini, Marom Bikson, Marco Iacoboni, David S. Liebeskind, Jason D. Hinman, S Thomas Carmichael, Jeffrey L. Saver

**Affiliations:** 1 Department of Neurology and Comprehensive Stroke Center, David Geffen School of Medicine at UCLA, 710 Westwood Plaza, Los Angeles, CA 90095 USA; 2 Department of Biomedical Engineering, The City College of New York (CCNY), New York, NY USA; 3 Department of Psychiatry and Biobehavioral Sciences, David Geffen School of Medicine at UCLA, Los Angeles, CA USA

**Keywords:** Translational readiness, Assessment tool, Neuroprotection, Acute cerebral ischemia

## Abstract

Many neuroprotective and other therapies for treatment of acute ischemic stroke have failed in translation to human studies, indicating a need for more rigorous, multidimensional quality assessment of the totality of preclinical evidence supporting a therapy prior to conducting human trials. A consensus panel of stroke preclinical model and human clinical trial experts assessed candidate items for the translational readiness scale, compiled from prior instruments (STAIR, ARRIVE, CAMARADES, RoB 2) based on importance, reliability, and feasibility. Once constructed, the tool was applied by two independent raters to four current candidate acute stroke therapies, including two pharmacologic agents [nerinetide and trans-sodium crocetinate] and two device interventions [cathodal transcranial direct current stimulation and fastigial nucleus stimulation]. The Preclinical evidence of Readiness In stroke Models Evaluating Drugs and Devices (PRIMED^2^) assessment tool rates the totality of evidence available from all reported preclinical animal stroke model studies in 11 domains related to diversity of tested animals, time windows, feasibility of agent route of delivery, and robustness of effect magnitude. Within each content domain, clearly operationalized rules assign strength of evidence ratings of 0–2. When applied to the four assessed candidate agents, inter-rater reliability was high (kappa = 0.88), and each agent showed a unique profile of evidentiary strengths and weaknesses. The PRIMED^2^ assessment tool provides a multidimensional assessment of the cumulative preclinical evidence for a candidate acute stroke therapy on factors judged important for successful basic-to-clinical translation. Further evaluation and refinement of this tool is desirable to improve successful translation of therapies for acute stroke.

## Introduction

Many neuroprotective and other therapies for treatment of acute ischemic stroke have failed in translation despite demonstrating apparent preclinical evidence of efficacy [1–5]. These failures have been attributed to deficiencies in the design and conduct of both preclinical studies and of human clinical trials. For preclinical studies, identified methodologic weaknesses in study quality include absence of randomization, lack of blinded assessment of outcome, failure to prespecify a primary endpoint, use of clinically unattainable treatment times, and publication bias [6–9].

To address these deficiencies, consensus groups have developed two categories of methods to assess the quality of preclinical acute stroke studies to increase the odds of translational success. The first category encompasses tools that only or primarily assess the quality of individual preclinical studies. These include (1) the Stroke Therapy Academic Industry Roundtable (STAIR) criteria; (2) the Animal Research: Reporting In Vivo Experiments (ARRIVE); and (3) the Preclinical Risk of Bias (P-RoB) assessment tool. The second category is tools that assess the quality of meta-analyses of preclinical studies. The leading example is the Collaborative Approach to Meta-Analysis and Review of Animal Data from Experimental Studies (CAMARADES) checklist. These tools have had a substantial beneficial effect on the preclinical literature, with the quality of studies improving over time [10].

However, in addition to these tools, there is a recognized need for an instrument that would complement and extend them by more broadly assessing a treatment’s readiness for translation from preclinical studies to initial human trials, as was highlighted by discussions at the 2020 STAIR XI meeting (October 2020 virtual conference) [11]. Such a tool would rate the strength of the cumulative evidence across all studies in the literature with regard to key aspects needed for treatment success, including effect reproducibility; studies across animals of different ages, sex, species, and comorbidities; presence of a dose–response effect; evaluation of feasible time windows and routes of delivery; and assessment of the magnitude of demonstrated beneficial imaging and behavioral effects. Ideally, the tool would also be designed for rapid assimilation both numerically and figurally. To address this need, we developed a novel assessment tool: the Preclinical evidence of Readiness In stroke Models Evaluating Drugs and Devices (PRIMED^2^) translational assessment rating system.

## Methods

The assessment tool was developed through consensus-building discussions, tool drafting, and iterative review and revision by the author group. The consensus panel was selected to include both experts in preclinical stroke models and experts in human stroke clinical trials.

To identify potential topic domains to be analyzed by the instrument, first, existing content items were compiled from three existing preclinical study assessments (STAIR criteria, ARRIVE, CAMARADES) and two existing assessments of human clinical trial quality—the Consolidated Standards of Reporting Trials (CONSORT) checklist and the Cochrane Collaboration Risk of Bias 2 (RoB 2) assessment tool. Experts were then asked to identify additional candidate topic domains not covered by the compilation from existing instruments. From this extended topic list, the group then identified a final set of domains to be assessed based on judgment of each domain’s importance, reliability, and feasibility. Importance is defined as the value of the domain in showing a genuine biological benefit of an agent. Reliability is defined as the capacity of the domain to be congruently measured by independent raters. Feasibility is defined based on the availability of the data for detailed review.

Next, within each content domain, strength of evidence ratings of 0–2 were developed with clearly operationalized rules for assigning strength scores. For visual presentation, the color coding of red, yellow, and green representing low, intermediate, and high strength was adapted from the RoB 2 tool scoring format.

To assess tool performance, the PRIMED^2^ tool was then applied by two independent raters to rate the cumulative preclinical literature regarding four candidate acute stroke therapies currently in development. Disagreements between raters were resolved by consensus discussion and inter-rater reliability assessed with the kappa statistic. The two pharmacologic interventions analyzed were nerinetide (NA1), a post-synaptic density-95 protein inhibitor; and trans-sodium crocetinate (TSC), an oxygen diffuser enhancer [12–20]. The two device interventions analyzed were cathodal transcranial direct current stimulation (C-tDCS), direct current applied to ischemic hemisphere with anti-excitatory and collateral enhancing effects; and deep cerebellar fastigial nucleus stimulation (FNS), activating cerebral blood flow enhancing and neuroprotective central cholinergic pathways [21–28]. These interventions were chosen from among agents in current development to assess applicability of the scale to diverse types of intervention. Accordingly, two of the treatments selected were drug agents and two were device interventions. In addition, the pharmacologic agents analyzed were chosen to have different mechanisms of action and the device interventions were selected to include an approach involving direct stimulation of the ischemic zone and an approach involving focal stimulation remote from the ischemic zone. Lastly, as a positive control, we also wished to include among the 4 assessed interventions a treatment that had successfully transitioned from demonstrated preclinical efficacy to demonstrated clinical efficacy to use in clinical practice [29]. However, unfortunately, no such agent has yet completed the full development pathway. We, therefore, selected, as one of the pharmalogic treatments, the currently investigated agent that has shown the strongest signal of potential beneficial effect in initial, completed human trials (nerinetide).

Data sharing is not applicable to this article as no datasets were generated during the current study.

## Results

After compilation and generation of candidate domains followed by prioritization based on importance, reliability, and feasibility, 11 assessment items were selected for final inclusion. The items characterize the diversity of animals in which benefit was demonstrated (4 items—age, sex, species, comorbidities); the time windows in which benefit was demonstrated (2 items—number of time epochs and attainability of times in clinical settings); the feasibility of agent route of delivery (1 item); and the robustness of effect magnitude (3 items—infarct volume, behavioral outcomes, and dose response).

With regard to operationalized rules for assigning scores of 0–2, four of the items had dichotomous responses and were assigned possible scores of 0 or 2. Seven of the items had graded responses and were assigned possible scores of 0, 1, and 2. For eight items, the operationalized rules could be stated clearly with brief phrases. For three items of treatment time epoch, feasible time window, and feasible route of delivery, more detailed text guidance was judged needed.

For the treatment timing item, five epochs were delineated based on the timing relation of treatment administration to start and stop of ischemia induction: (1) pre-ischemia onset administration (treatment start prior to or concurrent with ischemic period onset); (2) transient ischemia, post-onset administration (treatment starts after transient ischemia onset and treatment continuation throughout the ischemia period; (3) permanent ischemia, post-onset administration (treatment starts after permanent ischemia onset); (4) transient ischemia, post-reperfusion administration (treatment starts after the end of ischemic period). Absence of benefit in any time epoch is scored 0, presence of benefit in one time epoch scored 1, and presence of benefit in two or more time epochs scored 2.

For the feasible time window item, a feasible time window is defined as 45 min or more from ischemia onset (scored 2) and an infeasible time window as less than 45 min (scored 0). The 45-min cutpoint was used for the feasibility of time window definition because it is the earliest start time of therapy that has been achieved in a pivotal human acute stroke clinical trial [30].

To classify the effect size of observed infarct reductions, meta-analytic Cohen’s *d* values (preferred) or cross-study median Cohen’s *d* values (less preferred but more easily performed) are employed. Effect sizes are categorized as (a) small: Cohen’s *d* = 0.2–0.39; (b) medium: Cohen’s *d* = 0.4–0.69; or (c) large: Cohen’s *d* ≥ 0.7 [31, 32].

The separate scoring of each of the 11 PRIMED^2^ elements and their visual-figural display is the primary component of the instrument, arraying agent evidence along multiple dimensions. An optional summary score totaling all component items is provided, but with caution as it fails to convey the granular information of each scale item.

In applying the assessment tool to the preclinical literature regarding the four exemplar therapies, inter-rater reliability showed a kappa statistic of 0.88. The PRIMED^2^ scores and figural output for all four agents are shown in Table 1. For two items, treatment time epoch and infarct volume reduction magnitude, high scores of 2 were achieved by all four agents. Items with three agents rating at high scores of 2 were age of animals, reproducibility across species and laboratories, and feasible time window. Conversely, for one item, sex of animals, low scores of 0 were present for three of the four agents. For another item, baseline comorbidities, all four agents showed either low 0 or intermediate 1 scores. Each of the four agents showed distinctive profiles of evidence of readiness. High and moderately high total readiness for translation scores were achieved by the two pharmacologic agents. The two device interventions achieved low (deep FNS) and high (C-tDCS) intermediate scores.

**Table 1:**
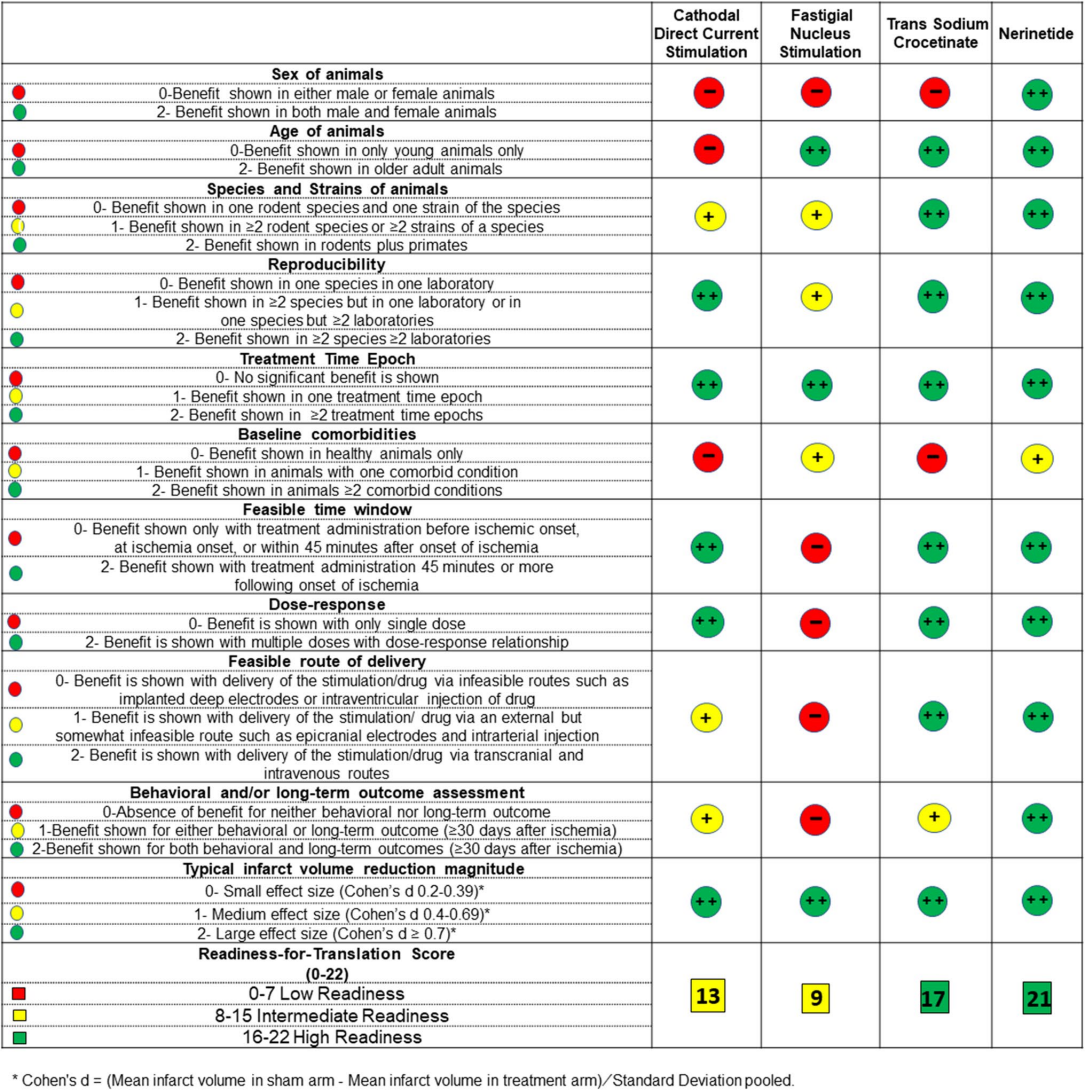
The PRIMED^2^ tool applied to 4 candidate neuroprotecive therapy agents. Color coding of red, yellow, and green represents low, intermediate, and high strength in each of the 11 areas assessed

## Discussion

The Preclinical evidence of Readiness In stroke Models Evaluating Drugs and Devices (PRIMED^2^) is a novel multidimensional tool assessing experimental acute stroke agent readiness to proceed to human clinical trial testing. For a candidate agent, the assessment instrument delineates the strength of the collective evidence from all preclinical studies in the literature in 11 domains judged important for translational success. These key domains have the capacity to be reliably rated and to be feasibly abstracted from published studies.

The PRIMED^2^ has several strengths. It is a multidimensional assessment evaluating the demographic, clinical, and species diversity of animals in which benefit has been shown. Also, the tool uses explicit, operationalized criteria to assign evidential strength ratings in each assessed domain. Furthermore, like the Cochrane RoB 2 instrument for human clinical trials, the PRIMED^2^ provides a visual-figural profile of evidence for an agent that is rapidly assimilable by readers and it is applicable to both drugs and device neuroprotective therapies. In addition, the instrument assesses magnitude of treatment effect with Cohen’s *d*, a standardized, unit-less effect size metric that accounts for study sample size and variability, and allows for the harmonization of studies that report absolute volume reduction and studies that report relative volume reduction [31, 32].

When applied to four current acute stroke agents in development, the PRIMED^2^ showed high inter-rater reliability. The instrument has good capacity to delineate evidence profiles, displaying unique patterns of item evidence for each of the four interventions. The PRIMED^2^ additionally demonstrated absence of floor and ceiling effects and good discriminative validity, assigning the four agents a wide range of overall scores.

The PRIMED^2^ tool differs from and complements available widely employed rating tools to assess preclinical stroke studies. Compared with the STAIR checklist, the PRIMED^2^ focuses solely upon rating the cumulative findings across all available studies, rather than a mix of items for rating individual studies and cumulative findings. It also provides a formal, operationalized, graded scoring system, whereas the STAIR checklist characterizes items qualitatively and somewhat dichotomously. These same features differentiate PRIMED^2^ from the ARRIVE checklist, with added distinction that PRIMED^2^ incorporates items specific to the acute stroke disease state while ARRIVE is a generic instrument with items applicable to all disease states and no stroke-specific domains. While the CAMARADES checklist is also a whole-literature assessment like PRIMED^2^, the current instrument incorporates items specific to the acute stroke disease state while CAMARADES is a generic instrument with items applicable to all disease states and no stroke-specific domains.

We have also provided an overall quantitative summary score option for the PRIMED^2^, but this total value should be used with caution as it can obscure the important granular information contained in the individual items. Final translational decision-making requires a detailed assessment of all the domains and their elements. A detailed assessment of all the tool’s domains particularly applies to the therapies that score in the intermediate readiness for translation range. The total score and individual items may also fail to reflect aspects of a therapy not assessable in animal models. For example, in this study, fastigial nucleus stimulation did not receive a high feasible route of delivery item score because all animal studies were performed by implantation of depth electrodes, an unattractive approach for human studies. However, in humans, non-invasive stimulation of deep structures is feasible, e.g., with focused ultrasound.

This study has limitations. The expert panel, though diverse in expertise, came from two institutions. Evaluation, and potentially revision, of the PRIMED^2^ by a larger, international consensus group is desirable. The PRIMED^2^ was applied to four agents currently in development; application to a broader set of interventions would be advantageous. The component items of the PRIMED^2^ tool are based on expert judgment of properties likely to be important to the successful clinical translation of acute stroke therapies, rather than empirically determined properties, as few agents have been successfully translated to date.

## Conclusion

The PRIMED^2^ assessment tool provides a multidimensional assessment of the cumulative preclinical evidence for a candidate acute stroke therapy on factors judged important for successful basic-to-clinical translation. Further evaluation and refinement of this tool is desirable to improve successful translation of therapies for acute stroke.

## Data Availability

Not applicable.
